# Scaling Across Environments: Distinct Genetic Architectures Underlie Thermal and Nutritional Plasticity of Size and Morphological Scaling in *Drosophila melanogaster*

**DOI:** 10.3390/biology15141157

**Published:** 2026-07-15

**Authors:** Shampa M. Ghosh, Isabelle M. Vea, Austin S. Wilcox, W. Anthony Frankino, Alexander W. Shingleton

**Affiliations:** 1 School of Biotechnology, Kalinga Institute of Industrial Technology (KIIT), Bhubaneswar 751024, India; shampa.ghosh@kiitbiotech.ac.in; 2 Department of Biological Sciences, University of Illinois Chicago, 840 W Taylor St., Chicago, IL 60607, USA; isabelle.vea@gmail.com (I.M.V.); aswilco2@uic.edu (A.S.W.); 3 Department of Biology and Biochemistry, University of Houston, Houston, TX 77204, USA; frankino@uh.edu

**Keywords:** allometry, body size, *Drosophila melanogaster*, genetic architecture, morphological scaling relationship, phenotypic plasticity, reaction norm, thermal plasticity, nutritional plasticity

## Abstract

Across animals, variation in adult body size is accompanied by coordinated variation in the size of individual morphological traits. However, the same morphological trait can scale differently with body size depending on what drives the size variation. In *Drosophila melanogaster*, for example, wing size scales differently from body size when size varies because of developmental nutrition versus developmental temperature. Whether the genetic basis of size plasticity and scaling is shared across different environmental regulators of size remains unclear, but it is central to predicting how selection acts on the developmental mechanisms that regulate trait size, plasticity, and morphological scaling. Using ~100 isogenic *D. melanogaster* lineages, we measured wing and leg size across nutritional and thermal treatments. For each lineage, we estimated nutritional and thermal plasticity for both traits, as well as the wing–leg individual-level scaling relationship, or ILSR, generated by each environmental source of size variation. We found extensive genetic variation in both thermal and nutritional plasticity for wings and legs and in the slope of the ILSR between them. However, a lineage’s thermal plasticity was genetically uncorrelated with its nutritional plasticity for either trait, and we detected no genetic correlation between the slopes of thermal and nutritional wing–leg ILSRs. We also found no genetic correlation in the slope of nutritional wing–leg ILSRs between temperatures. Thus, the slope of a lineage’s nutritional ILSR at 17 °C was not predictive of its slope at 25 °C or 28 °C. Nevertheless, the overall pattern of nutritional ILSRs was conserved across temperatures. These results suggest that the genetic architecture of size plasticity and scaling depends on the environmental source of size variation. Consequently, the evolutionary response of scaling to selection in heterogeneous environments may not be predictable from genetic variation measured in any single environment.

## Simple Summary

When animals grow larger, their body parts, such as wings and legs, usually grow larger too. But how much each part grows compared to overall body size can depend on what caused the size difference in the first place, such as how much food an animal had or how warm its environment was while developing. Scientists do not fully understand how these size relationships are inherited or how they evolve. In this study, the researchers raised many genetically distinct lines of fruit flies under different nutritional levels and temperature conditions and measured their wings and legs. They found that a fly line’s sensitivity to temperature was inherited separately from its sensitivity to nutrition: lines that changed size a lot with temperature did not necessarily change a lot with food and vice versa. The way wings and legs grew together also followed different rules for temperature and nutrition. This suggests that the biological machinery controlling growth responds to temperature and nutrition through largely independent genetic pathways. Understanding this matters, because it shows that how a trait evolves may depend heavily on the environment, making the evolution of animal shape harder to predict than previously thought.

## 1. Introduction

Variation in adult body size within populations is perhaps one of the most familiar forms of phenotypic variation and reflects the combined effects of genetic and environmental factors on growth and development. To maintain function, variation in body size must be accompanied by coordinated variation in the sizes of individual-level morphological traits. Thus, larger humans tend to have longer legs, arms, and torsos, bigger livers, and larger hearts. The resulting covariation in trait size among adults within a population is known as a static morphological scaling relationship (or static allometry) and defines the characteristic body shape of that population or species [1,2,3,4,5]. Because changes in organismal form are fundamentally changes in the relative sizes of morphological traits, the evolution of morphology can on first approximation be viewed as the evolution of scaling relationships [1,2,6]. Accordingly, morphological scaling has been a central topic in evolutionary and developmental biology for more than 150 years [7]. Despite this long history of research, the developmental and genetic mechanisms governing the evolution of scaling relationships remain poorly understood.

Morphological scaling relationships are typically represented as log-linear relationships between trait sizes, described by the linear allometric equation log *y = α log x + b*, where *x* and *y* are the sizes of individual-level traits, measured on the same scale [8]. Here, *α* (the allometric coefficient) describes the extent to which the size of *y* changes relative to *x* as both vary with overall body size, while *b* captures the relative size of the two traits at a reference size (log *x* = 0). The equation describes observed relationships between trait sizes without assuming any underlying causes. Nevertheless, the allometric coefficient reflects the extent to which variation in the size of trait *x* is accompanied by variation in the size of trait *y*, and thus captures the relative sensitivity of the two traits to the factors that generate size variation.

Importantly, the allometric coefficient for the scaling relationship between two traits can vary depending on the factor that generates size variation. For example, in *Drosophila melanogaster*, the allometric coefficient for the scaling relationship between wing and body size is ~1 when size varies due to nutritional variation during larval development (developmental nutrition), but ~1.6 when size varies due to developmental temperature during the same ontogenetic period [9]. For the leg, in contrast, the same coefficients are ~1 and ~0.6, respectively. If trait size were determined mechanistically by body size, then the allometric coefficient would be invariant across different environmental drivers of size. That this is not the case indicates that traits differ in their sensitivities to different factors that regulate size and that overall body size is a reflection of the aggregate of these sensitivities. Scaling relationships therefore emerge from how individual-level traits respond to growth-regulatory signals rather than representing fixed properties of the organism as a whole.

**Figure 1:**
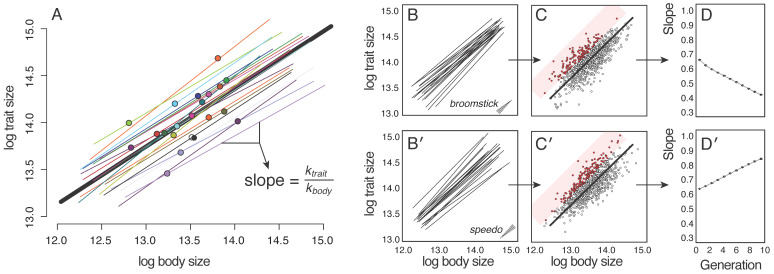
Population- and individual-level scaling relationships. (**A**) A population-level scaling relationship (PLSR: heavy gray line) is fitted across individuals (colored points), each of which sits on its own individual-level scaling relationship (ILSR: colored lines). The slope of an ILSR is the ratio of the sensitivities (*k*) of the two traits to the environmental or genetic factor that generates size variation. (**B**,**B′**) The pattern of ILSRs in a population determines how the PLSR responds to selection. Two populations can differ in this underlying pattern even when they share the same PLSR, here illustrated by a “broomstick” pattern, in which ILSRs fan out as they approach the origin (**B**), and a “speedo” pattern, in which they fan out from the origin (**B′**). (**C**,**C′**) Realized individuals from the two populations (points) generate statistically indistinguishable PLSRs (heavy gray line), but the same pattern of selection on relative trait size (red points) targets very different subsets of ILSRs in each. (**D**,**D′**) As a consequence, the same form of selection drives the slope of the PLSRs in opposite directions across generations: the slope decreases in the broomstick population (**D**) and increases in the speedo population (**D′**). Figure adapted from [11].

This distinction impacts how scaling relationships are interpreted. The scaling relationship observed in a natural population, referred to as a population-level scaling relationship (PLSR), is shaped by the collection of genetic and environmental factors that determine trait size in each individual (Figure 1A). Because individuals differ genetically and are subject to different developmental environments, a PLSR is a statistical summary of myriad individually unique developmental processes rather than the expression of a single mechanism. As such, a PLSR cannot directly reveal the genetic variation upon which selection acts to change scaling, since selection operates on growth and developmental processes within individuals, not on the PLSR itself. Problematically, this means that the pattern or form of selection that produces/favors a particular scaling relationship cannot be inferred from the pattern of scaling exhibited in a population [10].

To address this challenge, the concept of an individual-level scaling relationship (ILSR) has been developed [10,11,12]. ILSRs describe scaling between traits for a single genotype when they covary in response to a single size-affecting factor (Figure 1A). Individual-level scaling relationships are obtained by rearing individuals with a single genotype across a gradient of an environmental variable (an environmental ILSR) [9] or by introducing allelic variation at a single size-regulatory genetic locus (a genetic ILSR) [13]. The slope of an ILSR (*α*) is therefore controlled by the relative sensitivity of traits to the size-influencing factor (Figure 1A) and can be very different depending on what this factor is [9]. When the regulator is environmental, these sensitivities correspond to the environmental responsiveness of the traits, that is, their plasticity. Because the slope of an environmental ILSR captures the relative plasticity of two traits, it is this form of ILSR that we focus on here.

Empirically, ILSRs can only be generated in plants or animals that can be genetically replicated, such as clonal species or experimentally produced isogenic lineages. Consequently, for most species, ILSRs are cryptic, with the observed trait sizes being single points on unseen ILSRs, each ILSR generated by the individual’s genotypic response to a single size-regulatory factor (Figure 1A). Although these relationships are rarely directly observed (or even observable), their variation reflects genetic variation in the developmental mechanisms that co-regulate trait growth in response to a particular environmental factor. It is this variation, rather than the PLSR itself, upon which selection acts to change morphology.

Mathematical modeling of ILSRs reveals that their pattern dictates how a PLSR will respond to selection (Figure 1B) [10]. Because the pattern exhibited by ILSRs in morphospace reflects genetic variation in the relative sensitivities to growth-regulatory environmental factors among individuals, a form of GxE interaction, the pattern determines the direction and magnitude of evolutionary response of scaling to selection. Critically, the pattern of ILSRs cannot easily be inferred from the corresponding PLSR (Figure 1C). Thus, two populations may have ostensibly identical PLSRs while differing in the underlying pattern of ILSRs, reflecting genetic variation in their growth-regulating mechanisms. As a result, they may respond very differently to the same selective pressure (Figure 1D).

Despite their centrality to understanding the evolution of scaling relationships, few studies have described the pattern of ILSRs in a population [11,14]. Those that have done so have typically examined responses to a single environmental factor, most commonly nutrition. Although different environmental factors generate distinct ILSRs [9], it is not known whether the pattern of ILSRs that are generated in response to one environmental factor (for example, nutritional ILSRs) changes in response to a second environmental factor (for example, temperature), a form of (G × E_1_) × E_2_ interaction [15,16]. Certainly, PLSRs appear themselves be plastic: in horned beetles, for example, the body size–horn size allometry shifts with larval diet and across seasons [17,18]. The extent to which such shifts represent changes in the underlying pattern of ILSRs is unexplored. This is an important question: since the pattern of ILSRs in a population determines how the PLSR will respond to selection, environmentally induced changes in this pattern may cause the same selective pressure to produce different evolutionary changes in scaling under different environmental conditions.

Here, we determined if genotypes respond to variation in different environmental factors similarly or differently. Using multiple isogenic lineages of *Drosophila*, we quantified the effects of two environmental factors—temperature and nutrition—on (i) wing size, (ii) leg size, and (iii) the ILSR between them. Because ILSRs reflect relative trait size plasticity, we first investigated whether the plastic responses were genetically correlated between traits within environmental factors and within traits between environmental factors. We then tested whether the slope of the wing–leg ILSR was genetically correlated among environmental factors and whether this impacted the pattern of nutritional ILSRs at different temperatures.

## 2. Materials and Methods

All data and the R code used to analyze them are provided on Dryad (DOI: 10.5061/dryad.zpc866tqq). The phenotypic measurements of wing and leg size under starvation treatments were originally collected and described in Vea et al. [19]. No other data have been previously reported. During the preparation of this manuscript, the authors used generative AI (Claude Opus 4.8) to annotate the *R* code and improve the clarity and readability of the text.

### 2.1. Fly Stocks

All individuals analyzed in this study were drawn from 102 isogenic lineages from the Drosophila Genome Reference Panel (DGRP), a collection of approximately 200 fully sequenced, inbred *D. melanogaster* lines derived from a single natural population originally sampled in Raleigh, NC, USA [20]. Flies were reared on standard cornmeal–molasses medium [21] under a 12:12 h light–dark photoperiod at 75% humidity, unless otherwise stated.

### 2.2. Starvation Treatment

*Drosophila* egg collection, rearing, and phenotyping followed our established protocols [21,22,23]. For each DGRP lineage, females oviposited for three days. At 24 and 48 h, eggs were collected, divided into lots of 50, placed into vials containing 10 mL of standard cornmeal–molasses medium, and reared at 22 °C 12:12 L:D. This generated two age cohorts of flies (D0 and D1). When third-instar larvae from D0 began to pupariate, larvae from all cohorts were removed from the food and placed into empty food vials with a wet cotton plug to provide moisture. Pupae were removed from these vials and transferred to individual 1.5 mL Eppendorf tubes, each with a small hole in the lid, to complete development to adulthood. Larvae in the D0 cohort were starved for 0–24 h before pupariation, and larvae in the D1 cohort were starved for 24–48 h before pupariation. Because larvae stop feeding ~24 h before pupariation [24], D0 larvae were essentially allowed to feed ad libitum and more or less achieved full adult body size. In contrast, D1 larvae were starved before larval wandering, reducing adult size depending on their size at initiation of starvation. Across all cohorts, our starvation treatment therefore generated nutritionally induced variation in body size. We refer to D0 flies as fed and D1 flies as starved. Newly eclosed adults were collected for morphometric measurement.

### 2.3. Thermal Treatment

For each DGRP lineage, three replicate parental vials were established. Females in each vial were allowed to oviposit for 24 h until at least 50 eggs were deposited. Each vial was then assigned to one of three developmental temperatures (17 °C, 25 °C, or 28 °C). Eggs and larvae were allowed to develop at their assigned temperature until pupation and adult eclosion. Newly eclosed adults were collected for subsequent analysis.

### 2.4. Wing and Leg Size Measurement

Sex is a major determinant of both body size and body size plasticity in *D. melanogaster*. To reduce the number of confounding variables in our analyses, we therefore measured and analyzed trait size in male flies only. Trait sizes were measured using established protocols [9,22]. Briefly, *Drosophila* adults were dissected and their right wing and first right leg mounted in dimethyl hydantoin formaldehyde (DMHF). Wing area and femur length (a proxy for leg length [9]) were measured via semiautomated custom software (MetaMorph 7.10.2.240, Molecular Devices LLC, San Jose, CA, USA) that captures images from a digital camera-equipped microscope (Leica DM6000B, Leica Microsystems Inc., Deerfield, IL, USA). Femur length was squared to put it in the same dimension as wing area, and all measurements were log-transformed to ensure scale invariance across traits of different sizes.

### 2.5. Statistical Analysis

All analyses were conducted in *R* (v.4.5.1) [25], and the data and script to analyze them are provided on Dryad (https://doi.org/10.5061/dryad.zpc866tqq).

#### 2.5.1. Analysis of Thermal Versus Nutritional Plasticity and Scaling

A major challenge to analyzing genetic variation and covariation in plasticity and scaling is that both are characteristics of groups rather than individuals. One solution is to extract summary statistics from each genotype (for example, mean trait size under each environmental condition) and use these in subsequent tests of genetic correlation. However, these summary statistics do not capture the uncertainty of measurements within each lineage, which will lead to anti-conservative estimates of statistical significance when genetic correlations are calculated from indices derived from these estimates. To address this uncertainty, we first used the *MCMCglmm* package (v.2.36) in *R* [26] to generate 1000 independent estimates of lineage-specific trait sizes under each environmental condition (nutritional variation: fed vs. starved at 22 °C; thermal variation: fed at 17 °C, 25 °C, and 28 °C). Because the data for the effects of nutrition and temperature on trait size were collected in two separate experiments, they were analyzed separately. The specific models were:

(1) $$\mathrm{Nutrition}:\;y_{t,i,j,k}=\mu_{t}+\beta_{t}D_{i}+a_{t,j}+b_{t,i,j}D_{i}+\varepsilon_{t,i,j,k}$$

where $y_{t,i,j,k}$ is the size of trait *t* for individual *k* from lineage *j* reared in condition *i*, $\mu_{t}$ is the population mean for trait *t* (wing or leg) in fed conditions, $\beta_{t}$ is the population-level effect of starvation on trait *t*, $D_{i}$ is an indicator variable (fed: $D_{i}=$ 0; starved: $D_{i}=$ 1), $a_{t,j}$ is the random deviation of lineage *j* from the mean population size, $b_{t,i,j}$ is the random deviation of lineage *j* from the population-level plasticity, and $\varepsilon_{t,i,j,k}$ is random error.

(2) $$\mathrm{Temperature}:\;y_{t,i,j,k}=\mu_{t}+\beta_{t,17}T17_{i}+\beta_{t,28}T28_{i}+a_{t,j}+b_{t,17,j}\;T17_{i}+b_{t,28,j}\;T28_{i}+\varepsilon_{t,i,j,k}$$

where $\beta_{t,17}$ and $\beta_{t,28}$ are the population-level effects of 17 °C and 28 °C relative to 25 °C on trait *t*, *T*17*_i_* and *T*28*_i_* are indicator variables, and $b_{t,17,j}$ and $b_{t,28,j}$ are the corresponding lineage-specific deviations. Population-level thermal plasticity between 17 °C and 28 °C is $\beta_{t,28}-\beta_{t,17}$, and lineage-specific thermal plasticity is $\left(\beta_{t,28}+b_{t,28,j}\right)-\left(\beta_{t,17}+b_{t,17,j}\right)$.

Each model was fitted in *MCMCglmm* using parameter-expanded inverse-Wishart priors: for the lineage random effect, we placed an inverse-Wishart prior on the 4 × 4 covariance matrix of trait-specific intercepts and slopes (**V** = 0.02·**I**_4_, ν = 4), and for the residual structure an inverse-Wishart prior on the 2 × 2 cross-trait covariance (**V** = **I**_2_, ν = 2). We ran a single chain for 260,000 iterations, discarded the first 60,000 as burn-in, and thinned the remainder at an interval of 200, yielding 1000 stored posterior samples per parameter. Convergence and mixing were assessed by visual inspection of trace plots, by confirming that the lag-1 autocorrelation of the thinned chains was below 0.1 for all parameters, and by verifying that effective sample sizes were 515–1890 (close to 1000 for most parameters). The posterior distributions of all key correlations and scaling slopes were unimodal and approximately symmetric (Supplementary Figure S1).

For each lineage, we extracted 1000 posterior draws of three quantities from each model: (**i**) trait size under each environmental condition (wing and leg size in fed and starved conditions from the nutrition model; wing and leg size at 17 °C, 25 °C, and 28 °C from the temperature model); (**ii**) trait plasticity (the environmental effect size, β*_t_* + *b_t,j_* for each trait); and (**iii**) the slope of the ILSR between wing and leg size when they covary in response to the same environmental factor. All ILSRs were fitted using major axis (MA) regression, and the slope was estimated as the ratio of the wing and leg plasticity values within each environmental shift (fed → starved; 17–25 °C; 25–28 °C; 17–28 °C) (see Supplementary Methods for justification). ILSRs were only estimated for lineages where the 95% HPD interval of the plasticity estimates excluded zero; that is, where there was a demonstrable effect of the environment on trait size.

We then used these draws in subsequent analysis of genetic variance, covariance, and correlations within and among lineages for trait size, plasticity, and scaling. For correlations of plasticity and scaling between environmental treatments, where nutritional and thermal plasticity were estimated from separate models, we computed the cross-lineage correlation for each draw, pairing draws by index across models. Correlations among lineages were visualized using MA regression.

Throughout these analyses, we recorded the model value of the summary statistics based on the 1000 posterior draws along with their 95% highest posterior density (HPD) interval. For all plots, we present the 95% HPD interval of the MA along with the modal values for each lineage. The 95% HPD intervals not overlapping with zero were used to indicate strong evidence of effect.

#### 2.5.2. Analysis of Within-Temperature Plasticity and Scaling

To estimate plasticity and scaling in wing and leg size within each temperature, we used a frequentist approach rather than the Bayesian approach used above, because the quantities of interest are more tractable to estimate and test outside a hierarchical Bayesian model.

To estimate variance in wing and leg size within each temperature, we fitted the temperature model (Equation (2)) using the *glmmTMB* package (v.1.1.14) in *R* [27], which allowed us to model temperature-specific residuals. We estimated within-lineage variance from the residual dispersion component of the fitted model and the among-lineage variance at each temperature from the lineage variance–covariance matrix. The proportion of variance that could be attributed to within-lineage variation in size at each temperature was calculated as the residual variance divided by the total variance. To test whether this variance was significant, we compared models in which the residual dispersion was either constant or allowed to vary across temperatures.

To test whether the slope of the within-temperature wing–leg ILSRs changed systematically with temperature, we used a bootstrap procedure that accounted for estimation uncertainty in the per-lineage slopes. For each of 1000 bootstrap replicates, we resampled wing and leg measurements within each lineage–temperature combination with replacement and calculated the slope of the MA regression of wing on leg. We then fitted a fixed-effect model of slope in response to temperature (categorical factor), with lineage as a fixed factor, to isolate within-lineage changes across temperatures rather than variation among lineages. For each resample, we calculated the change in slope from 17 °C to 25 °C (δ_17.25_) and from 25 °C to 28 °C (δ_25.28_). Two-tailed *p* values for δ_17.25_ and δ_25.28_ were calculated as twice the smaller of the proportion of the 1000 bootstrap estimates above and below zero. We used a similar bootstrap approach to determine whether there was a genetic correlation in the slope of the within-temperature wing–leg ILSR between 17 °C and 25 °C and between 25 °C and 28 °C. Because the slope of an MA regression can become unstable when the correlation between *x* and *y* is low, we only conducted analyses of within-temperature scaling on the lineages with >5 individuals per lineage per temperature and where the correlation between wing and leg size *r_wing,leg_* > 0.3. To confirm the results were not sensitive to filter stringency, we repeated these analyses with no filter (all lineages included) and with a more stringent filter (>10 individuals per lineage per temperature and *r_wing,leg_* > 0.5).

We also tested whether there was random (rather than systematic) variation in the effect of temperature on the slope of the wing–leg ILSR by comparing two mixed linear models using a likelihood ratio test:

(3) $$\mathrm{Model}\;1:\;w_{i,j,k}=\mu +\beta_{leg}l_{i,j,k}+\beta_{T}T_{i}+\beta_{leg\cdot T}l_{i,j,k}T_{i}+a_{j}+b_{j}l_{i,j,k}+\varepsilon_{i,j,k}$$

(4) $$\mathrm{Model}\;2:\;w_{i,j,k}=\mu +\beta_{leg}l_{i,j,k}+\beta_{T}T_{i}+\beta_{leg\cdot T}l_{i,j,k}T_{i}+a_{j}+b_{j}l_{i,j,k}T_{i}+\varepsilon_{i,j,k}$$

where $w_{i,j,k}$ is wing size of individual k from lineage j at temperature i, $l_{i,j,k}$ is leg size, and $T_{i}$ is temperature. Model 1 allows random lineage-specific deviation from the average wing–leg ILSR slope ($b_{j}l_{i,j,k}$) that is constant across temperatures. Model 2 allows random lineage-specific deviation from the average wing–leg ILSR slope ($b_{j}l_{i,j,k}T_{i}$) that varies with temperatures. Thus, Model 2 allows each lineage to respond differently to temperature with respect to the effect of temperature on wing–leg ILSR slope changes. The models were fitted using the *lme4* package(v.1.1-37) in *R* [28], and the conditional *R*^2^ for each model was estimated using *MuMIn* package (v.1.48.19) [29].

To determine the pattern of within-temperature ILSR, we fitted an MA regression to wing on leg size in each lineage at each temperature and calculated the median point of intersection (MPI) among ILSRs [10] and the bivariate mean of wing and leg size across lineages.

## 3. Results

### 3.1. There Is Extensive Genetic Variation in Nutritional and Thermal Size Plasticity of Wing and Leg

We measured wing and leg size in 102 isogenic lineages subjected to both nutritional and thermal variation during development. We measured an average of 63 flies per lineage for the nutritional treatment (range: 13–498) and 51 flies per lineage for the thermal treatment (range: 10–104).

**Figure 2:**
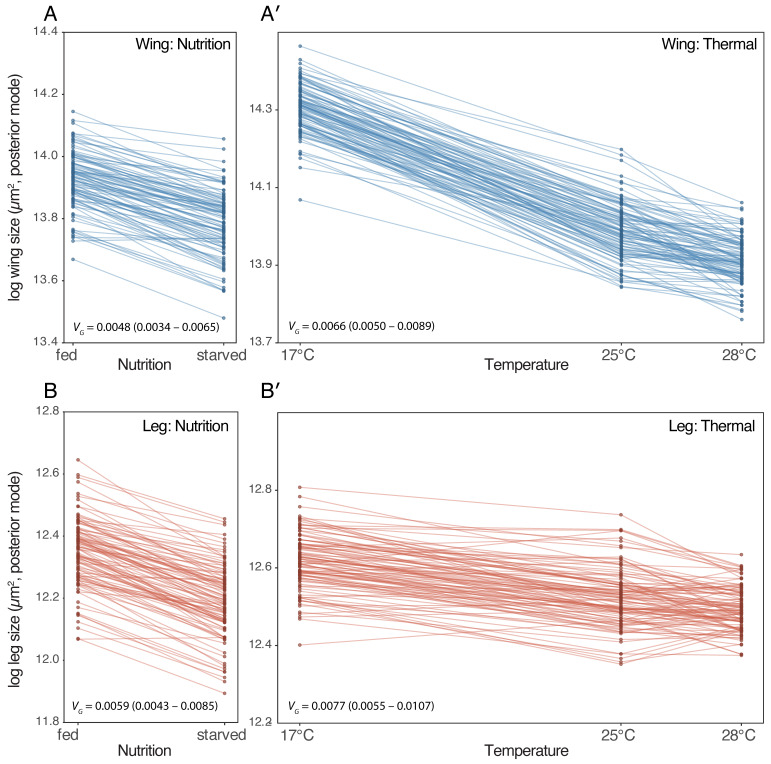
Genetic variation in the reaction norms of wing and leg size against nutrition and temperature. (**A**) Nutritional and (**A′**) thermal plasticity of wing size. (**B**) Nutritional and (**B′**) thermal plasticity of leg size. Points are posterior modes for each lineage across 1000 draws of the MCMCglm model. Note that the *y*-axes are on different scales between environments, but the same scale within.

Both wing and leg size responded strongly to diet and temperature, but the magnitude of that response varied substantially among DGRP lineages. There was considerable genetic variation among lineages in both thermal and nutritional size plasticity (GxE) of wing and leg, with the 95% HPD interval for *V_GxE_* excluding zero in all cases (Figure 2, Supplementary Figure S2). Consistent with previous studies [9], wing size was considerably more thermally plastic than leg size: the difference in thermal plasticity between the wing and the leg had a mode of −0.276 (95% HPD: −0.298, −0.256). In contrast, the leg was marginally more nutritionally plastic than the wing (differences in nutritional plasticity, mode = 0.021, 95% HPD interval: 0.007, 0.031).

Plasticity varied among genotypes both independently of and in proportion to overall trait sizes. Previous authors have used a cross-environment genetic correlation of <1 as an indicator of genetic variation in plasticity [30,31], and for both traits across all environmental shifts (e.g., fed → starved; 17 °C → 25 °C, etc.), this correlation was <1 (Supplementary Table S1). It is important to note, however, that a genetic correlation of less than 1 is a sufficient, but not necessary indicator of genetic variation in plasticity of trait size. If the genetic correlation for trait size between environments = 1, but the slope of that relationship differs from 1, plasticity varies among genotypes, albeit systematically with overall trait size (Supplementary Figure S3). For most cross-environment relationships, the slope of the relationship differed from 1 (Supplementary Table S1), indicating that trait plasticity either increased or decreased with overall size, although not consistently in direction.

### 3.2. Thermal and Nutritional Plasticity Are Not Genetically Correlated

**Figure 3:**
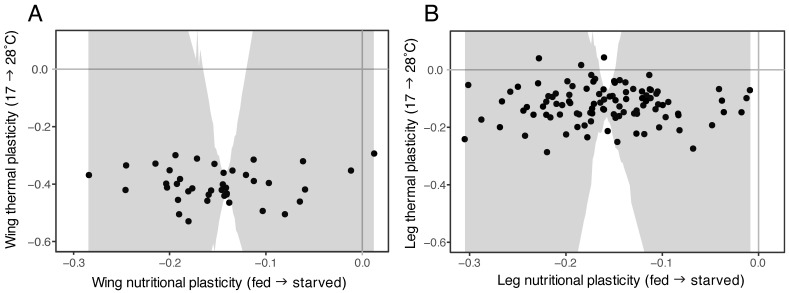
There is no genetic correlation between thermal and nutritional plasticity within traits. (**A**) Relationship between thermal and nutritional plasticity of wing size. (**B**) Relationship between thermal and nutritional plasticity of leg size. Points are modal values of plasticity estimates per lineage. The HPDs (gray) for the correlation between nutritional and thermal plasticity contained zero for both wing and leg (Supplementary Table S2).

A lineage’s sensitivity to temperature was genetically independent of its sensitivity to nutrition. Our data strongly support a genetic correlation between the plasticity of wing size and plasticity of leg size as long as both are responding to the same environmental variable (fed → starved; 17 °C → 25 °C; 25 °C → 28 °C; 17 °C → 28 °C, Supplementary Table S2). In contrast, we found no evidence that thermal plasticity is genetically correlated with nutritional plasticity for either trait (Figure 3): in all cases, the 95% HPD intervals for the correlations contained zero (Supplementary Table S2).

There was considerable genetic variation in the shape of the thermal reaction norms for both the wing and the leg. For both traits, the data strongly supported a negative correlation in plasticity between the 17 °C → 25 °C and 25 °C → 28 °C intervals (Supplementary Table S2): lineages that shrank the most between 17 °C and 25 °C shrank the least between 25 °C and 28 °C and vice versa. This is consistent with the thermal reaction norms being nonlinear across the full temperature range, but where they converge at the temperature extremes (17 °C and 28 °C) and diverge at intermediate temperatures (25 °C) (Figure 2).

### 3.3. Thermal and Nutritional Scaling Relationships Are Not Genetically Correlated

**Figure 4:**
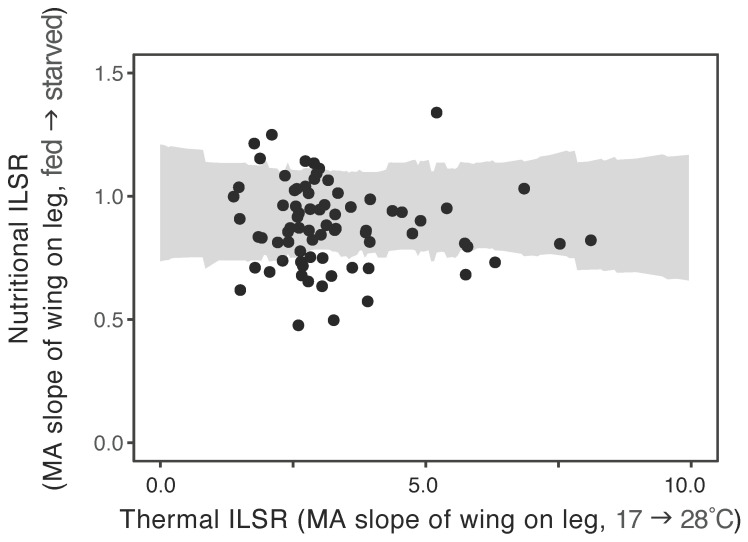
There is no genetic correlation between the thermal and nutritional ILSRs for wing against leg size. Points are modal values of scaling estimates per lineage. The HPD (gray) for the correlation between nutritional and thermal scaling contains zero (Supplementary Tables S3 and S4).

Although we found no evidence that size plasticity is correlated across different environmental regulators of size, this does not preclude genetic correlations in the scaling relationship across different environmental axes. That is, lineages with a steeper wing–leg scaling relationship when size varies with nutrition (nutritional ILSRs) may also show a steeper wing–leg scaling relationship when size varies with temperature (thermal ILSRs). However, our data did not support this hypothesis (Figure 4): in all cases, the 95% HPD intervals for the correlations for the slope of wing–leg ILSRs between different environmental regulators of size contained zero (Supplementary Table S3). This conclusion was robust to the choice of correlation method: a rank-based (Spearman) correlation calculated within each posterior draw again returned 95% HPD intervals containing zero in all cases (Supplementary Table S4).

### 3.4. Nutritional Plasticity Changes with Temperature

The observation that scaling relationships generated by different environmental factors are not genetically correlated is consistent with previous studies that show that the pattern of scaling is different when body size varies in response to nutrition versus temperature [9]. These data suggest that the developmental mechanisms that regulate scaling in response to variation in temperature are independent of those that regulate scaling in response to variation in nutrition. If true, then we would expect nutritional scaling to be invariant across temperatures and vice versa.

Testing this prediction requires measurement of the nutritional scaling relationship at different temperatures. Although we did not explicitly impose variation in nutrition at different temperatures in our experiment, 30–61% of the variation in trait size across lineages and temperatures could be attributed to within-lineage variation, i.e., variation in trait size among genetically identical individuals reared on the same food at the same temperature. There are many potential sources of this variation, including developmental noise [32], maternal effects [33], and within-vial microenvironmental effects [34]. A major factor, however, is competition for nutrition, which is impacted by the distribution of larval age [35] and density [36]. We therefore hypothesized that within-lineage variation in size was primarily mediated by nutrition. To test this, we calculated the angle between the within-lineage wing:leg ILSR and that lineage’s nutritional and thermal ILSRs (Supplementary Figure S4). Across all lineages, we found that the within-lineage ILSR aligned with the nutritional ILSR, as measured using our diet manipulation. We therefore concluded that within-temperature variation in trait size for a lineage could be used as a proxy for a lineage’s nutrition plasticity and the within-temperature MA regression of wing on leg size as a proxy for the nutritional ILSR of the two traits at that temperature.

Temperature systematically affected the magnitude of nutritional plasticity across lineages. Within-lineage nutritional plasticity differed across temperatures for both traits (leg: χ^2^_2_ = 43.54, *p* < 0.0001; wing: χ^2^_2_ = 7.55, *p* = 0.023), increasing between 25 °C and 28 °C for the wing (glmmTMB, z = 2.36, *p* = 0.018) and between 17 °C and 25 °C for the leg (glmmTMB, z = 4.87, *p* < 0.0001). Thus, there is a (G × E_1_) × E_2_ effect on the size of traits such that a lineage’s response to variation in nutrition depends on temperature.

### 3.5. Nutritional Scaling Does Not Change Consistently with Temperature

Across lineages, the slope of the wing–leg nutritional ILSR showed no consistent directional change with temperature (two-tailed bootstrap *p*-value, δ_17.25_ = −1.43, *p* = 0.61; δ_25.28_ = 1.40, *p* = 0.50). Because the MA slope became unpredictable when correlations were low, this analysis was restricted to the 62 lineages with *r_wing,leg_* > 0.3 and *n* > 5 at all three temperatures, although the result was robust across a range of filter conditions (Supplementary Table S5). Temperature therefore appears to affect the overall magnitude of nutritional plasticity without consistently changing how plastic the wing is relative to the leg.

This does not mean slope was unaffected by temperature, but rather that its response was lineage-specific rather than shared. A model of wing against leg size allowing the temperature effect on slope to vary among lineages (Model 2) fitted the data slightly, but significantly better than one in which temperature acted uniformly (Model 1) (conditional *R*^2^ = 0.957 for Model 2 vs 0.941 for Model 1; likelihood-ratio test: χ^2^ = 1218, *N* = 5100, df = 7, *p* < 2.2 × 10^−16^). Consistent with this, slopes were uncorrelated across lineages between temperatures (two-tailed bootstrap *p*-value, 17 °C vs. 25 °C: *r* = 0.009, *p* = 0.94; 25 °C vs. 28 °C: *r* = 0.075, *p* = 0.53): a lineage with a steep slope at one temperature was no more likely to have a steep slope at another. These results were again robust across a range of filter conditions (Supplementary Table S6).

Together, the absence of any shared directional change in slope and the lack of genetic correlation in slope between temperatures indicate that temperature alters individual lineages’ nutritional scaling idiosyncratically, with no common direction. This is consistent with the hypothesis that thermal and nutritional scaling is largely governed by independent mechanisms.

### 3.6. The Pattern of Individual-Level Scaling Relationships Does Not Change with Temperature

**Figure 5:**
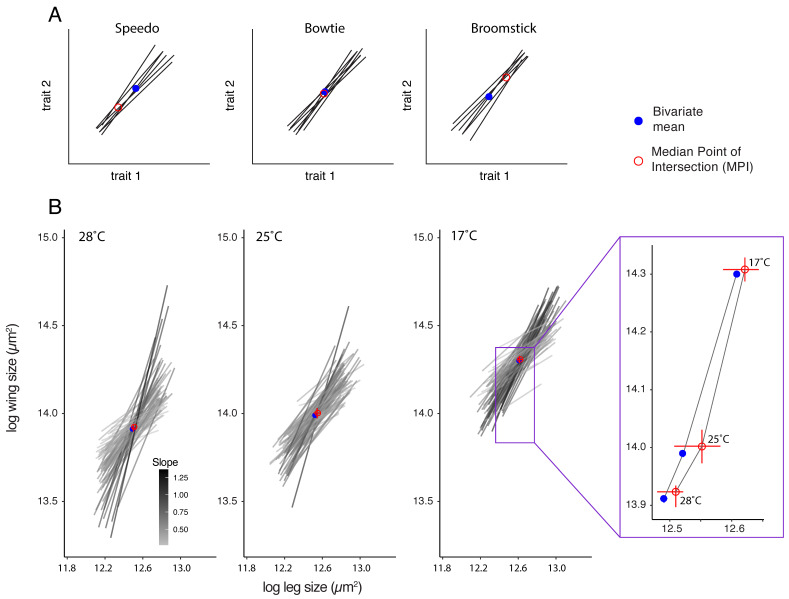
The pattern of the nutritional individual-level scaling relationships does not change with temperature. (**A**) The pattern of ILSRs is categorized by the position of the median point of intersection (MPI, open red circle) among ILSRs relative to the bivariate mean of trait size (closed blue circle), generating speedo, bowtie, or broomstick distributions. (**B**) At all three temperatures, the pattern of nutritional ILSRs is more or less a bowtie. Inset shows the position of the bivariate mean of wing and leg size and the median point of intersection at each temperature. Error bars are 95% HPD and are hidden in some cases.

Our previous work demonstrated that the pattern of ILSRs in a population determines how the PLSR should respond to selection. Although we did not find any consistent change in individual-level nutritional scaling across temperatures, it remains possible that the pattern of ILSRs is temperature-sensitive. This pattern is broadly defined by the relationship between the median point of intersection (MPI) among ILSRs and the bivariate mean of the traits: if the bivariate mean and the MPI intersect, the pattern is *bowtie*; if the bivariate mean is farther from the origin than the MPI, the pattern is *speedo*; and if the bivariate mean is closer to the origin than the MPI, the pattern is *broomstick* (Figure 5A). Both the MPI and bivariate means shifted with temperature, but remained approximately coincident (Figure 5B), suggesting that the pattern of ILSRs is bowtie across temperatures.

## 4. Discussion

The pattern of individual-level scaling relationships (ILSRs) in a population is typically hidden, but is predicted to determine the response of the population-level scaling relationship (PLSR) to selection [10]. Because different traits differ in their sensitivity to different environmental regulators of size, ILSRs may vary depending on the environmental factor generating size variation. What is unclear is whether this impacts the pattern of ILSRs in a population, and if so, the nature and consequence of this impact. Using temperature and nutrition as different generators of size variation, we found no genetic correlation between thermally and nutritionally induced size plasticity for a single trait, no genetic correlation between the slope of thermal and nutritional ILSRs, and no detectable change in the pattern of nutritional ILSRs under different thermal conditions. While there is some evidence that nutritional plasticity increases with temperature for both the wing and the leg, these data indicate that thermal and nutritional scaling are largely genetically decoupled. This is consistent with the developmental–genetic mechanisms regulating thermal scaling being at least partly independent of those regulating nutritional scaling, although this would need to be confirmed with functional–genetic approaches.

Historically, the study of phenotypic plasticity, both at proximate-developmental and ultimate-evolutionary levels, has primarily focused on the effect of a single environmental variable. Nevertheless, there is an increasing appreciation that organisms develop in multidimensional environments, generating multidimensional plastic responses [15,16,37,38]. The interaction of temperature with other environmental regulators of size, including nutrition, has perhaps received the most attention [39,40,41,42,43,44,45]. In *D. melanogaster*, the general trend appears to be an increase in nutritional plasticity of trait size with an increase in temperature [46], with higher temperatures exacerbating the negative effects of a poor diet [39]. Our data are consistent with this observation, indicating that nutritional plasticity increases with temperature in both the wing and leg in *D. melanogaster*.

While our data support an interaction between the effects of temperature and nutrition on trait size, we did not find evidence that this systematically influenced the slope of the wing–leg ILSR across temperatures. Further, the pattern of the nutritional ILSRs did not change between 17 °C, 25 °C, and 28 °C, with the MPI and the wing–leg bivariate mean maintaining their position relative to each other across temperatures. Consequently, although the position of nutritional ILSRs shifted toward the origin with increasing temperature, the pattern of these relationships was thermally insensitive (Figure 5B).

The constancy of the pattern of nutritional ILSRs across temperatures has important implications for the mode of selection that is predicted to alter the slope of PLSRs. Because the pattern of ILSRs for a population is predicted to determine the phenotypic response of the PLSR to selection [10], if this pattern is thermally insensitive, the same form of selection will generate the same response regardless of the thermal environment at which individuals develop. For example, selection on increased or decreased relative wing size (wing–thorax ratio) in *D. melanogaster* increases and decreases the slope of the wing–thorax scaling relationship at 25 °C, respectively [47]. This is presumably because selection targets individuals that have correspondingly steep or shallow nutritional wing–thorax ILSRs [10]. Our data suggest that the population will respond in the same way regardless of the temperature at which selection is being applied.

Although the same form of selection on nutritional scaling may generate the same response at different temperatures, however, the genotypes contributing to that response may differ. This is because we found no genetic correlation in the slope of nutritional ILSRs across temperatures: a lineage with a relatively steep wing–leg scaling relationship at 17 °C did not necessarily exhibit a similarly steep relationship at 25 °C. This was also evident as a significant random interaction between temperature and lineage on slopes such that ILSR slopes changed with temperature, but differently for different lineages. The result is a higher-order (G × E_1_) × E_2_ interaction, in which the genetic architecture underlying variation in nutritional scaling (G × E_1_)—i.e., which genotypes contribute high-slope and low-slope phenotypes—is itself a function of the temperature (E_2_) at which nutritionally induced size variation is generated. Consequently, the phenotypic response to selection at one temperature may have a different genetic basis for the same phenotypic response to selection produced at another temperature. Such (G × E_1_) × E_2_ interactions substantially complicate predictions of how traits respond to selection in heterogeneous environments [15,48,49].

There are several caveats to the interpretation of our data. First, we studied only males. The slope and intercept of the wing–body and leg–body nutritional ILSRs are slightly, but significantly different for females and males [14]. It is possible, therefore, that the slope of wing–leg ILSR is genetically correlated across temperatures in females, but not in males. The pattern of nutritional ILSRs is also slightly different in males and females [14], and this pattern may also change with temperature in females, but not in males. However, the slope of the wing–body and leg–body ILSRs are genetically correlated between the sexes [14], so our findings in males likely extend to females. Regardless, additional experiments are needed to explore the effect of sex on the pattern of ILSRs in *D. melanogaster* and how this influences the effects of selection on ILSRs and the consequences for the PLSR.

A second caveat is that we did not explicitly impose variation in nutrition within our temperature treatments (17 °C, 25 °C and 28 °C), in contrast to our experiments exploring nutritional scaling at 22 °C. This variation may be due to a number of factors, including developmental noise, microenvironmental variation within vials, maternal effects, and competition for nutrition due to crowding effects and variation in larval age. The observation that a lineage’s within-temperature wing–leg ILSR aligns with its nutritional ILSR (Supplementary Figure S3), however, suggests that the phenotypic responses to these factors converge on the same physiological and developmental mechanisms as nutritional plasticity. Nevertheless, size variation was more limited within thermal treatments. This reduces the precision of the MA regressions to estimate the slopes of ILSRs, expanding the confidence limits around our estimates. This in turn reduces the power of our analyses to capture genetic correlation between slopes, and may explain why we did not observe a genetic correlation in the slope of ILSRs across temperatures. Future experiments directly manipulating developmental nutrition at temperatures other than 22 °C are necessary to more systematically test the hypothesis that ILSRs change with temperature.

The preceding limitation applies only to detecting changes in nutritional scaling across temperatures, however. Our primary conclusion that thermal and nutritional plasticity and scaling are not genetically correlated rests on firmer data. This is because thermal and nutritional effects were each estimated from large contrasts (between temperatures and between diets, respectively) and generated tightly bound posteriors (Supplementary Figures S1 and S2). Further, these same data were sufficient to detect genetic correlations between wing and leg plasticity, confirming that they had the power to detect genetic correlations where they existed. The absence of a genetic correlation between thermal and nutritional plasticity and scaling is therefore biologically meaningful rather than a limitation of power: if there were a correlation it would be weak and not change our conclusion that the genetic architectures of thermal and nutritional plasticity and scaling are largely independent.

## 5. Conclusions

In conclusion, our data are consistent with previous studies that suggest that nutritional and thermal scaling relationships are generated by largely distinct developmental and physiological processes. Further, our data suggest the hypothesis that even if the phenotypic response to selection on scaling is the same across environments, the genetic basis of this response differs. A compelling method to test this hypothesis would be to impose artificial selection to change the slope of nutritional scaling relationships at two different temperatures. If our hypothesis were correct, the populations should show the same response in their PLSR, but this response should be due to selection on different alleles such that the populations diverge in their genetic composition even as they converge in their phenotypes. Whether this is because different alleles target the same genes, signaling pathways, or physiological mechanisms in the two populations is an open question. More generally, our findings indicate that the genetic basis of plasticity and scaling evolution in heterogeneous environments cannot be inferred from heritability or selection response measured in any single environment. Because scaling is the substrate of morphological evolution and because the genetic architecture of scaling appears to vary across environments, the evolution of morphology in nature may be both richer and less predictable than single-environment studies suggest.

## Data Availability

All data and the R code used to analyze them are provided on Dryad: https://doi.org/10.5061/dryad.zpc866tqq.

## Supplementary Materials

The following supporting information can be downloaded at https://www.mdpi.com/article/10.3390/biology15141157/s1. Supplementary Methods; Supplementary Figure S1: Posterior distributions of the key cross-environment scaling correlations and wing–leg scaling slopes; Supplementary Figure S2: Plasticity in wing and leg size in response to nutrition (fed vs. starved) and temperature (17 °C vs. 28 °C) among *Drosophila* lineages, ranked within each panel from most to least plastic; Supplementary Figure S3: The genetic correlation among lineages of wing size in fed and starved flies; Supplementary Figure S4: Within-temperature scaling relationships align with nutritional scaling relationships rather than thermal scaling relationships across lineages. Supplementary Table S1: Genetic variances (diagonal, blue), genetic covariances (upper triangle, purple), and slopes of the MA regression ^†^ of trait size in one environment on trait size in another across lineages (lower triangle, orange) for wing and leg size—values shown as posterior mode (95% HPD interval); Supplementary Table S2: Genetic correlations among degrees of plasticity within and between traits; Supplementary Table S3: Genetic correlations among slopes of ILSRs generated by different environmental factors; Supplementary Table S4: Rank-base (Spearman) correlations among slopes of ILSRs generated by different environmental factors; Supplementary Table S5. Effect of changing filtering stringency on tests of the relationship between the slope of the within-temperature wing–leg nutritional ILSR and temperature; Supplementary Table S6. Effect of changing filtering stringency on the genetic correlation of within-temperature wing–leg nutritional ILSR slopes across temperatures. Reference [50] is cited in the Supplementary Materials.

## Abbreviations

The following abbreviations are used in this manuscript.

PLSR	population-level scaling relationship
ILSR	individual-level scaling relationship

## References

[1] Shingleton A.W. (2010). Allometry: The Study of Biological Scaling. Nat. Educ. Knowl..

[2] Gould S.J. (1966). Allometry and Size in Ontogeny and Phylogeny. Biol. Rev..

[3] Klingenberg C., Zimmermann M. (1992). Static, Ontogenic, and Evolutionary Allometry—A Multivariate Comparison in 9 Species of Water-Striders. Am. Nat..

[4] Stern D.L., Emlen D.J. (1999). The Developmental Basis for Allometry in Insects. Development.

[5] Shingleton A.W., Frankino W.A., Flatt T., Nijhout H.F., Emlen D.J. (2007). Size and Shape: The Developmental Regulation of Static Allometry in Insects. BioEssays.

[6] Pelabon C., Firmat C., Bolstad G.H., Voje K.L., Houle D., Cassara J., Rouzic A.L., Hansen T.F. (2014). Evolution of Morphological Allometry. Ann. N. Y. Acad. Sci..

[7] Gayon J. (2000). History of the Concept of Allometry. Am. Zool..

[8] Huxley J., Teissier G. (1936). Terminology of Relative Growth. Nature.

[9] Shingleton A.W., Estep C.M., Driscoll M.V., Dworkin I. (2009). Many Ways to Be Small: Different Environmental Regulators of Size Generate Distinct Scaling Relationships in *Drosophila melanogaster*. Proc. R. Soc. B Biol. Sci..

[10] Dreyer A.P., Ziabari O.S., Swanson E.M., Chawla A., Frankino W.A., Shingleton A.W. (2016). Cryptic Individual Scaling Relationships and the Evolution of Morphological Scaling. Evolution.

[11] Houle D., Jones L.T., Fortune R., Sztepanacz J.L. (2019). Why Does Allometry Evolve so Slowly?. Integr. Comp. Biol..

[12] Nijhout H.F., German R.Z. (2012). Developmental Causes of Allometry: New Models and Implications for Phenotypic Plasticity and Evolution. Integr. Comp. Biol..

[13] Pavlicev M., Norgard E.A., Fawcett G.L., Cheverud J.M. (2011). Evolution of Pleiotropy: Epistatic Interaction Pattern Supports a Mechanistic Model Underlying Variation in Genotype-Phenotype Map. J. Exp. Zool. Part B Mol. Dev. Evol..

[14] Wilcox A.S., Vea I.M., Frankino W.A., Shingleton A.W. (2023). Genetic Variation of Morphological Scaling in *Drosophila melanogaster*. Heredity.

[15] Westneat D.F., Potts L.J., Sasser K.L., Shaffer J.D. (2019). Causes and Consequences of Phenotypic Plasticity in Complex Environments. Trends Ecol. Evol..

[16] Hudak A., Dybdahl M. (2023). Phenotypic Plasticity under the Effects of Multiple Environmental Variables. Evolution.

[17] Moczek A.P. (2002). Allometric Plasticity in a Polyphenic Beetle. Ecol. Èntomol..

[18] Emlen D. (1997). Diet Alters Male Horn Allometry in the Beetle *Onthophagus acuminatus* (Coleoptera: Scarabaeidae). Proc. R. Soc. B Biol. Sci..

[19] Vea I.M., Wilcox A.S., Frankino W.A., Shingleton A.W. (2023). Genetic Variation in Sexual Size Dimorphism is Associated with Variation in Sex-Specific Plasticity in *Drosophila*. Am. Nat..

[20] Mackay T.F.C., Richards S., Stone E.A., Barbadilla A., Ayroles J.F., Zhu D., Casillas S., Han Y., Magwire M.M., Cridland J.M. (2012). The *Drosophila melanogaster* Genetic Reference Panel. Nature.

[21] Frankino W.A., Bakota E., Dworkin I., Wilkinson G.S., Wolf J.B., Shingleton A.W. (2019). Individual Cryptic Scaling Relationships and the Evolution of Animal Form. Integr. Comp. Biol..

[22] Stillwell R.C., Dworkin I., Shingleton A.W., Frankino W.A. (2011). Experimental Manipulation of Body Size to Estimate Morphological Scaling Relationships in *Drosophila*. J. Vis. Exp..

[23] Stillwell R.C., Shingleton A.W., Dworkin I., Frankino W.A. (2016). Tipping the Scales: Evolution of the Allometric Slope Independent of Average Trait Size. Evolution.

[24] Testa N.D., Ghosh S.M., Shingleton A.W. (2013). Sex-Specific Weight Loss Mediates Sexual Size Dimorphism in *Drosophila melanogaster*. PLoS ONE.

[25] R Core Team (2025). R: A Language and Environment for Statistical Computing.

[26] Hadfield J.D. (2010). MCMC Methods for Multi-Response Generalized Linear Mixed Models: The MCMCglmm R Package. J. Stat. Softw..

[27] Brooks M.E., Kristensen K., van Benthem K.J., Magnusson A., Berg C.W., Nielsen A., Skaug H.J., Mächler M., Bolker B.M. (2017). glmmTMB Balances Speed and Flexibility Among Packages for Zero-Inflated Generalized Linear Mixed Modeling. R J..

[28] Bates D., Mächler M., Bolker B., Walker S. (2015). Fitting Linear Mixed-Effects Models Using Lme4. J. Stat. Softw..

[29] Nakagawa S., Johnson P.C.D., Schielzeth H. (2017). The Coefficient of Determination R^2^ and Intra-Class Correlation Coefficient from Generalized Linear Mixed-Effects Models Revisited and Expanded. J. R. Soc. Interface.

[30] Via S., Lande R. (1985). Genotype-environment interaction and the evolution of phenotypic plasticity. Evolution.

[31] Choy Y.M.M., Walter G.M., Mirth C.K., Sgrò C.M. (2024). Within-Population Plastic Responses to Combined Thermal-Nutritional Stress Differ from Those in Response to Single Stressors, and Are Genetically Independent across Traits in Both Males and Females. J. Evol. Biol..

[32] Saito K., Tsuboi M., Takahashi Y. (2024). Developmental Noise and Phenotypic Plasticity Are Correlated in *Drosophila simulans*. Evol. Lett..

[33] Valtonen T.M., Kangassalo K., Pölkki M., Rantala M.J. (2012). Transgenerational Effects of Parental Larval Diet on Offspring Development Time, Adult Body Size and Pathogen Resistance in *Drosophila melanogaster*. PLoS ONE.

[34] Lin Y., Chen Z.-X., Oliver B., Harbison S.T. (2016). Microenvironmental Gene Expression Plasticity Among Individual *Drosophila melanogaster*. G3 Genes Genomes Genet..

[35] Venkitachalam S., Deep A., Das S., Joshi A. (2023). The Role of Greater Competitive Ability in Countering Age Disadvantages in Larval Competition in *Drosophila melanogaster*. bioRxiv.

[36] Klepsatel P., Procházka E., Gáliková M. (2018). Crowding of *Drosophila* Larvae Affects Lifespan and Other Life-History Traits via Reduced Availability of Dietary Yeast. Exp. Gerontol..

[37] Relyea R.A. (2004). Fine-tuned phenotypes: Tadpole plasticity under 16 combinations of predators and competitors. Ecology.

[38] Rodrigues Y.K., Beldade P. (2020). Thermal Plasticity in Insects’ Response to Climate Change and to Multifactorial Environments. Front. Ecol. Evol..

[39] Kutz T.C., Sgrò C.M., Mirth C.K. (2019). Interacting with Change: Diet Mediates How Larvae Respond to Their Thermal Environment. Funct. Ecol..

[40] Lee K.P., Roh C. (2010). Temperature-by-nutrient Interactions Affecting Growth Rate in an Insect Ectotherm. Entomol. Exp. Appl..

[41] Kingsolver J.G., Shlichta J.G., Ragland G.J., Massie K.R. (2006). Thermal Reaction Norms for Caterpillar Growth Depend on Diet. Evol. Ecol. Res..

[42] Stillwell R.C., Wallin W.G., Hitchcock L.J., Fox C.W. (2007). Phenotypic Plasticity in a Complex World: Interactive Effects of Food and Temperature on Fitness Components of a Seed Beetle. Oecologia.

[43] Chakraborty A., Sgrò C.M., Mirth C.K. (2025). Untangling Plastic Responses to Combined Thermal and Dietary Stress in Insects. Curr. Opin. Insect Sci..

[44] Clissold F.J., Simpson S.J. (2015). Temperature, Food Quality and Life History Traits of Herbivorous Insects. Curr. Opin. Insect Sci..

[45] Kim K.E., Jang T., Lee K.P. (2020). Combined Effects of Temperature and Macronutrient Balance on Life-History Traits in *Drosophila melanogaster*: Implications for Life-History Trade-Offs and Fundamental Niche. Oecologia.

[46] Chakraborty A., Walter G.M., Monro K., Alves A.N., Mirth C.K., Sgrò C.M. (2023). Within-population Variation in Body Size Plasticity in Response to Combined Nutritional and Thermal Stress is Partially Independent from Variation in Development Time. J. Evol. Biol..

[47] Robertson F.W. (1962). Changing the Relative Size of the Body Parts of *Drosophila* by Selection. Genet. Res..

[48] Sgrò C.M., Hoffmann A.A. (2004). Genetic Correlations, Tradeoffs and Environmental Variation. Heredity.

[49] Stinchcombe J.R., Kirkpatrick M., Grp F.T.W. (2012). Genetics and Evolution of Function-Valued Traits: Understanding Environmentally Responsive Phenotypes. Trends Ecol. Evol..

[50] Shingleton A.W. (2019). Symposium Article: Which Line to Follow? The Utility of Different Line-Fitting Methods to Capture the Mechanism of Morphological Scaling. Integr. Comp. Biol..

