# Supplementary material for: Scaling Across Environments: Distinct Genetic Architectures Underlie Thermal and Nutritional Plasticity of Size and Morphological Scaling in Drosophila melanogaster
Source: Biology (Basel). 2026 Jul 15;15(14):1157. doi: 10.3390/biology15141157 (PMC13405782; doi:10.3390/biology15141157)
Supplement: Supplementary file 1 [file biology-15-01157-s001.zip › biology-4398859-supplementary.pdf]

Supplementary Materials

Supplementary Methods

The scaling relationship between wing and leg size across an environmental gradient can be estimated using an MA regression [50]. We calculated the slope of the scaling relationship using the mean wing and leg size for each lineage in two environmental conditions (fed – starved; 17˚C – 25˚C; 25˚C – 28˚C; 17˚C – 28˚C). The MA slope of the relationship is calculated as:

$$slope = \frac{S_{yy} - S_{xx} + \sqrt{(S_{yy} - S_{xx})^2 + 4S_{xy}^2}}{2S_{xy}} \quad \text{(S1)}$$

For two points (e.g. wing and leg size in fed versus starved flies):

$$\bar{x} = \frac{x_1 + x_2}{2}, \bar{y} = \frac{y_1 + y_2}{2} \quad \text{(S2)}$$

And

$$S_{xx} = \frac{(x_1\text{-}x_2)^2}{4}, S_{yy} = \frac{(y_1\text{-}y_2)^2}{4}, S_{xy} = \frac{(x_1 - x_2)(y_1 - y_2)}{4} \quad \text{(S3)}$$

Inserting into Eqn S1:

$$b = \frac{(y_1 - y_2)^2 - (x_1 - x_2)^2 + \sqrt{[(y_1 - y_2)^2 - (x_1 - x_2)^2]^2 + 4(x_1 - x_2)^2(y_1 - y_2)^2}}{2(x_1 - x_2)(y_1 - y_2)} \quad \text{(S4)}$$

Simplifying:

$$b = \frac{y_1 - y_2}{x_1 - x_2} \quad \text{(S5)}$$

Thus the slope of the MA regression can be estimated as the ratio of the wing and leg plasticities across an environmental gradient.

50. Shingleton, A.W. Symposium Article: Which Line to Follow? The Utility of Different Line-Fitting Methods to Capture the Mechanism of Morphological Scaling. *Integr Comp Biol* **2019**, *59*, 1399–1410, doi:10.1093/icb/icz059.

Supplementary Figures

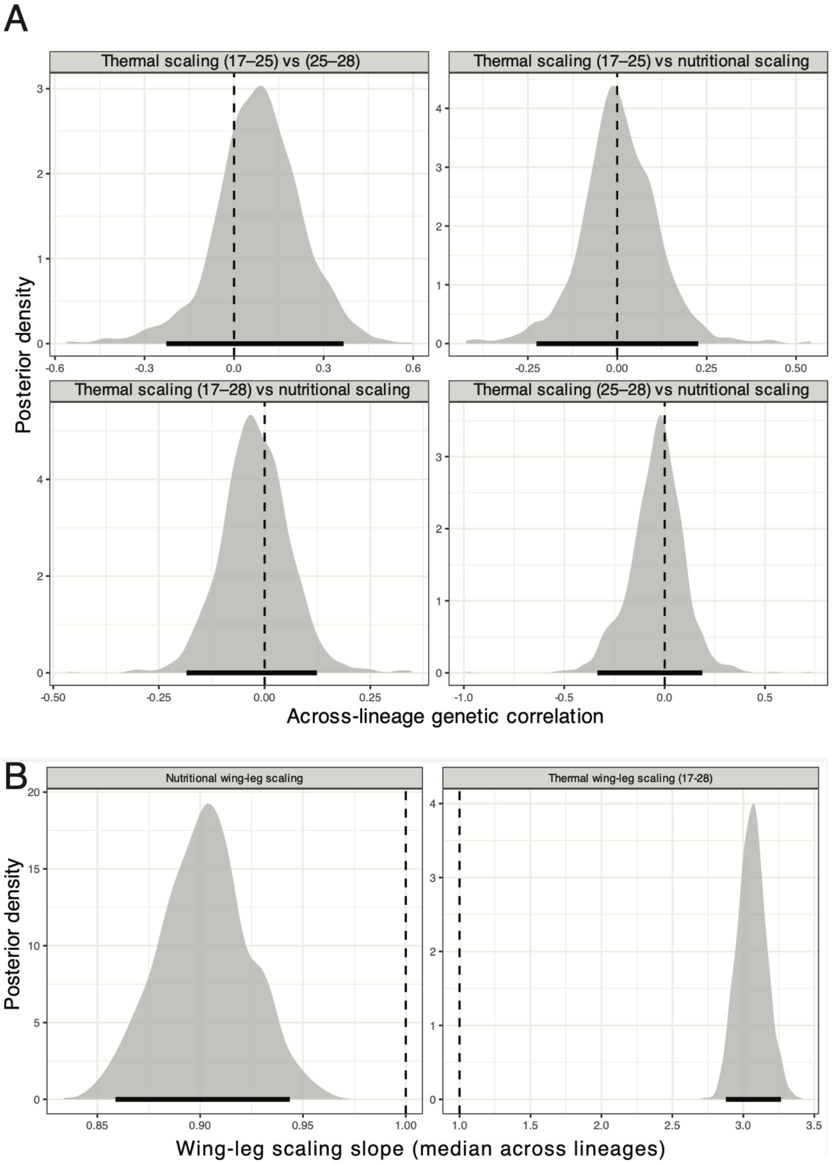

Supplementary Figure S1: Posterior distributions of the key across-environment scaling correlations and wing–leg scaling slopes. All distributions are derived from the posterior draws of the *MCMCglmm* models (1,000 draws per parameter); in each panel the shaded curve is the posterior density, the solid horizontal bar is the 95% highest posterior density (HPD) interval, and the dashed vertical line marks the null value. (A) Posterior distribution of the genetic correlation (*r*) between different wing-leg ILSRs. (B) Posterior distribution of the median slope of the nutritional and thermal wing-leg ILSR, across lineages..

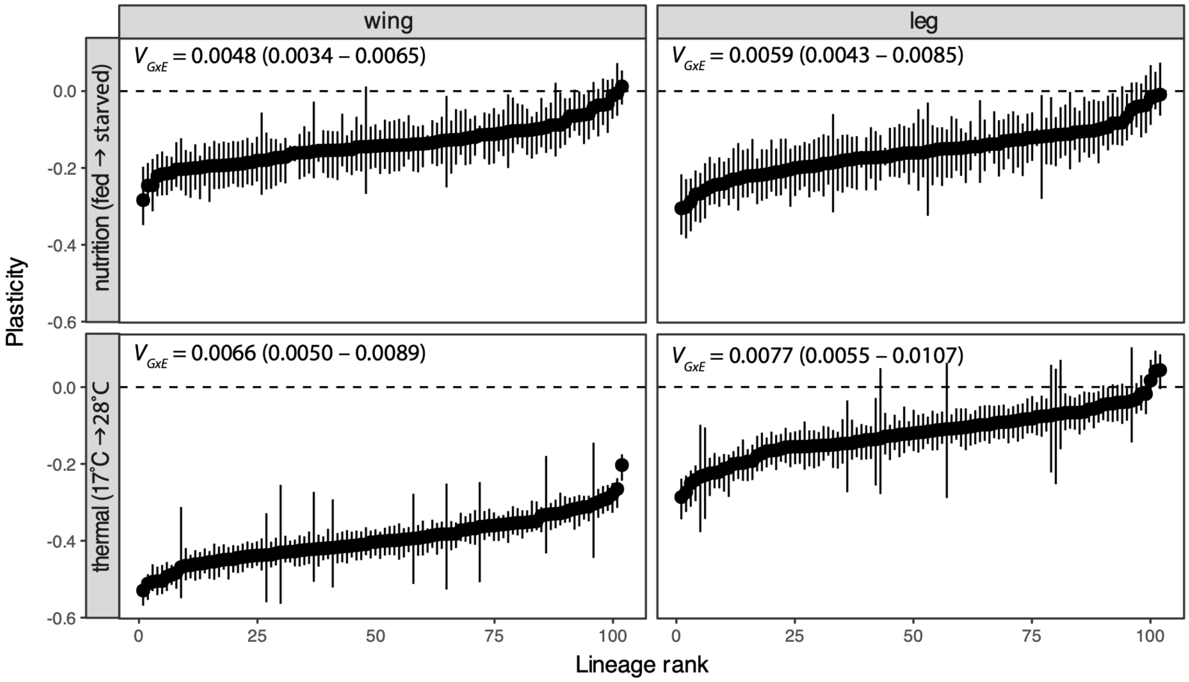

Supplementary Figure S2: Plasticity in wing and leg size in response to nutrition (fed vs. starved) and temperature (17 ˚C vs 28°C) among *Drosophila* lineages, ranked within each panel from most to least plastic. Points show the posterior mode with 95% HPD intervals. Almost all values are negative, reflecting reduced size under low nutrition or high temperature. V_g = modal genetic variance with 95% HPD intervals.

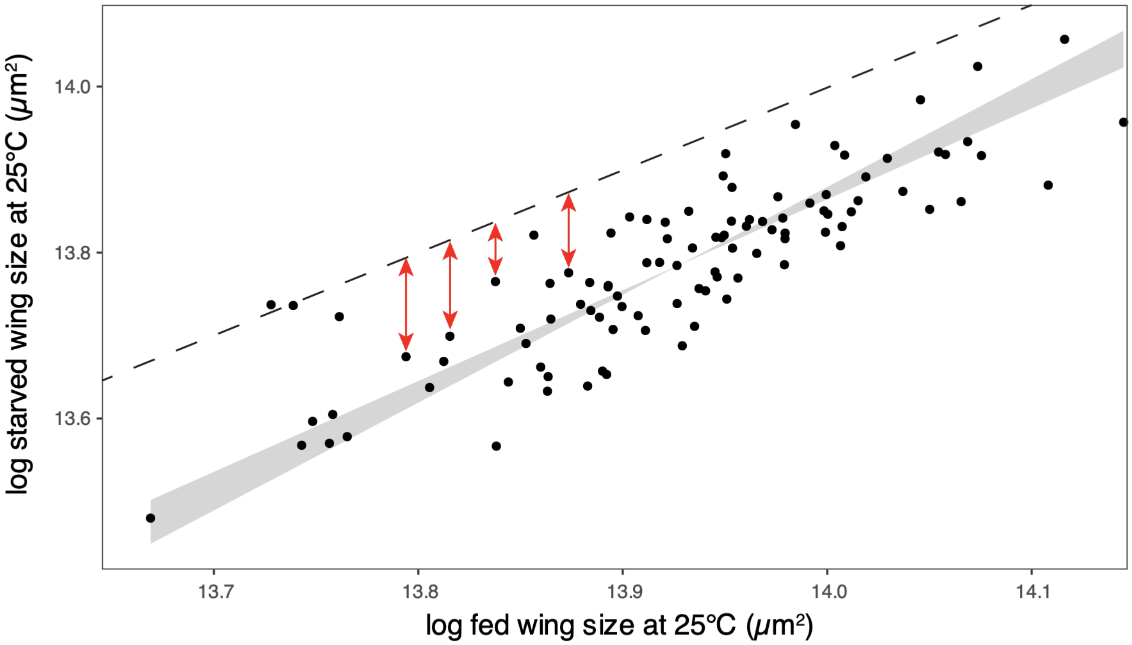

Supplementary Figure S3: The genetic correlation among lineages of wing size in fed and starved flies. The modal genetic correlation is 0.8144 (95% HPD interval: 0.7675–0.8463) consistent with genetic variation in nutritional plasticity. Broken line represents where wing size is the same in both fed and starved flies and red arrows show lineage-specific plasticity. Because the slope of the regression is >1 (1.2034, 95% HPD interval:1.0948–1.2994), plasticity decreases as overall wing size increases. This would be true even if the genetic correlation were 1, indicating that a genetic correlation of < 1 is a sufficient but not necessary indicator of genetic variation in plasticity (GxE). Gray shading shows the 95% HPD interval for the MA regression of starved on fed wing size.

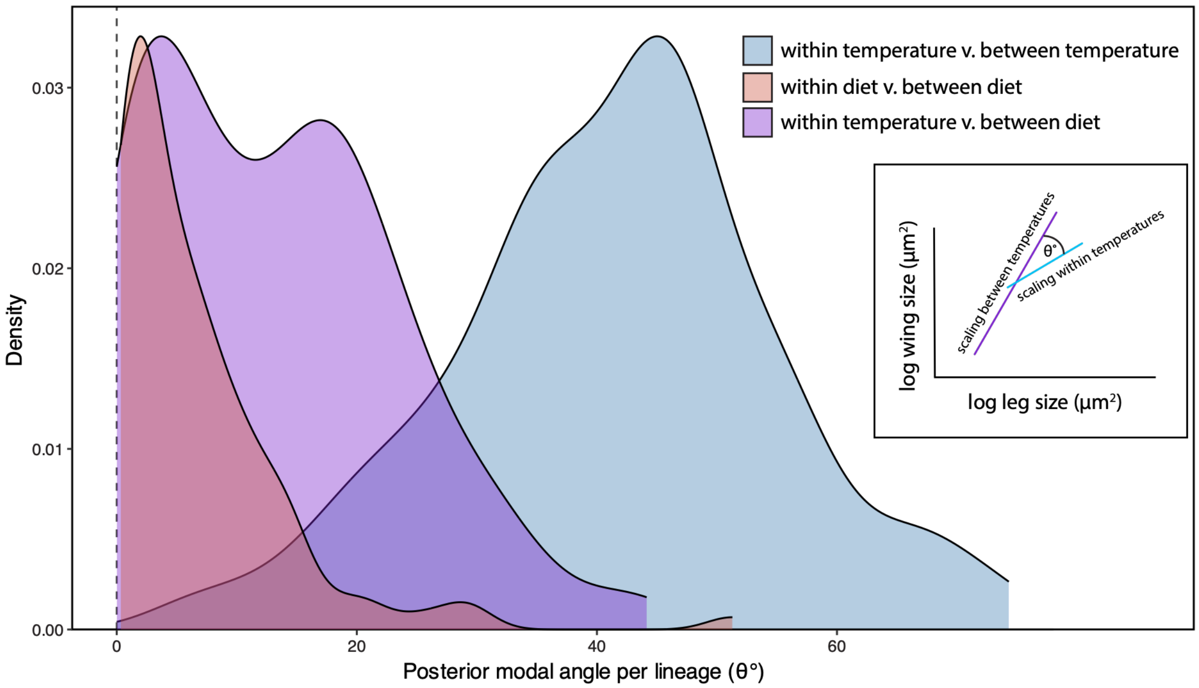

Supplementary Figure S4: Within-temperature scaling relationships align with nutritional scaling relationships rather than thermal scaling relationships across lineages. For each lineage, we extracted two axes: Axis 1: the within-temperature scaling relationship, defined as the first eigenvector of the within-temperature wing-leg covariance, averaged across temperatures; Axis 2: the thermal/nutritional scaling relationship, defined as the first eigenvector of the covariance of predicted mean wing and leg size across temperatures/diets from the posterior draw. We then quantified the angle between these two axes per lineage for each posterior draws (inset), which reflects the degree to which each lineage's thermal/nutritional scaling relationship is aligned with its within-temperature scaling relationship. An angle near 0° indicates that within-temperature scaling relationship for a lineage aligns with that lineage's thermal/nutritional scaling relationship. The distribution is the modal angle across draws for each lineage.

Supplementary Tables

Supplementary Table S1: Genetic variances (diagonal, blue), genetic covariances (upper triangle, purple), and slopes of the MA regression† of trait size in one environment on trait size in another across lineages (lower triangle, orange), for wing and leg size; values shown as posterior mode (95% HPD interval).

		**17˚C**	**25˚C**	**28˚C**	**Fed**	**Starved**
Wing	**17˚C**	0.0042 (0.0037, 0.0046)	0.5714 (0.4852, 0.6350)	0.5041 (0.3891, 0.5812)		
	**25˚C**	1.3518 (1.1819, 1.4994)	0.0056 (0.0052, 0.0064)	0.6675 (0.6020, 0.7158)		
	**28˚C**	1.0419 (1.0948, 1.2994)	0.6892 (0.5782, 0.7682)	0.0042 (0.0037, 0.0049)		
	**Fed**				0.0090 (0.0081,0.0098)	0.8144 (0.7675,0.8463)
	**Starved**				1.2034 (1.0948, 1.2994)	0.0018 (0.0106, 0.0130)

		**17˚C**	**25˚C**	**28˚C**	**Fed**	**Starved**
Leg	**17˚C**	0.0054 (0.0050, 0.0062)	0.6161 (0.5582, 0.6745)	0.4318 (0.3319, 0.5251)		
	**25˚C**	1.0913 (0.9692, 1.2462)	0.0061 (0.0056, 0.0069)	0.6586 (0.5987, 0.7248)		
	**28˚C**	0.6454 (0.4884, 0.7759)	0.6892 (0.5782, 0.7759)	0.0036 (0.0032, 0.0043)		
	**Fed**				0.0129 (0.0119, 0.0143)	0.8125 (0.7683, 0.8476)
	**Starved**				1.0846 (0.9676, 1.1579(	0.014 (0.0130, 0.0160)

† Slope of the major axis regression of trait size in the row environment (*y*) on trait size in the column environment (*x*), across lineages. For each regression, the trait size in the column environment (*x*) is typically larger than the trait size in the row environment (*y*). Thus if the slope is > 1, the plasticity increases with overall trait size, while if slope is <1, plasticity decreases with overall trait size (see Supplementary Figure S1).

Supplementary Table S2: Genetic correlation among plasticities within and between traits.

	Comparison	r[1]	95% HPD[2]
Wing vs Leg (same condition)	Nutritional plasticity	**0.85**	**(0.79, 0.89)**
	Thermal plasticity (17→25˚C)	**0.63**	**(0.53, 0.68)**
	Thermal plasticity (25→28˚C)	**0.58**	**(0.49, 0.67)**
	Thermal plasticity (17→28˚C)	**0.50**	**(0.35, 0.56)**
Within wing	Thermal (17→25˚C) vs (25→28˚C)	**-0.46**	**(-0.55, -0.35)**
	Nutrition vs thermal (17→25˚C)	0.12	(-0.01, 0.22)
	Nutrition vs thermal (25→28˚C)	-0.08	(-0.19, 0.06)
	Nutrition vs thermal (17→28˚C)	0.06	(-0.08, 0.19)
Within leg	Thermal (17→25 ˚C) vs (25→28˚C)	**-0.36**	**(-0.47, -0.21)**
	Nutrition vs thermal (17→25˚C)	0.00	(-0.09, 0.16)
	Nutrition vs thermal (25→28˚C)	-0.03	(-0.14, 0.12)
	Nutrition vs thermal (17→28˚C)	0.00	(-0.11, 0.15)

[1] Correlation coefficient
[2] 95% HPD. Intervals that exclude 0 are shown in **bold**

Supplementary Table S3: Genetic correlation among slopes of ILSRs generated by different environmental factors.

	Comparison	n	r[1]	95% HPD[2]
Thermal vs nutritional scaling	Thermal (17→25˚C) vs nutritional	69	-0.01	(-0.23, 0.23)
	Thermal (17→28˚C) vs nutritional	77	-0.04	(-0.18, 0.12)
	Thermal (25→28˚C) vs nutritional	27	-0.02	(-0.34, 0.19)
Thermal vs thermal scaling	Thermal (17→25˚C) vs (25→28˚C)	19	0.11	(-0.23, 0.37)

[1] Correlation coefficient
[2] 95% HPD. Intervals that exclude 0 are shown in **bold**

Supplementary Table S4: Rank-base (Spearman) correlation among slopes of ILSRs generated by different environmental factors.

	Comparison	n	r_s[1]	95% HPD[2]
Thermal vs nutritional scaling	Thermal (17→25˚C) vs nutritional	69	0.00	(-0.23, 0.16)
	Thermal (17→28˚C) vs nutritional	77	-0.05	(-0.25, 0.10)
	Thermal (25→28˚C) vs nutritional	27	-0.13	(-0.44, 0.17)
Thermal vs thermal scaling	Thermal (17→25˚C) vs (25→28˚C)	19	0.14	(-0.25, 0.46)

[1] Correlation coefficient
[2] 95% HPD. Intervals that exclude 0 are shown in **bold**

Supplementary Table S5. Effect of changing filtering stringency on tests of the relationship between the slope of the within-temperature wing-leg nutritional ILSR and temperature.

Filter Stringency[1]	Number of lineages analysed	$\delta_{17\cdot25}$ (*P*-value)[2]	$\delta_{25\cdot28}$ (*P*-value)33
n>10, *r* >0.5	23	0.063 (0.807)	0.315 (0.817)
None	102	0.507 (0.768)	0.206 (0.67)

[1] Criteria used to include lineages in the analysis. *n* = minimum number of individuals per lineage per temperature. *r* = minimum correlation coefficient for wing-leg relationship per lineage per temperature.
[2] Mean change in ILSR slope from 17 ˚C to 25 ˚C, across 1000 bootstrap replicates. *P*-value shown in parenthesis.
[3] Mean change in ILSR slope from 25 ˚C to 28 ˚C, across 1000 bootstrap replicates. *P*-value shown in parenthesis.

Supplementary Table S6. Effect of changing filtering stringency on the genetic correlation of within-temperature wing-leg nutritional ILSR slopes across temperatures.

Filter Stringency[1]	Number of lineages analysed	$r_{17\cdot25}$ (*P*-value)[2]	$r_{25\cdot28}$ (*P*-value)	$r_{17\cdot28}$(*P*-value)
n>10, *r* >0.5	23	-0.045 (0.760)	0.009 (0.588)	-.0.41 (0.754)
None	102	0.032 (0.848)	0.018 (0.8858)	0.021 (0.708)

[1] Criteria used to include lineages in the analysis. *n* = minimum number of individuals per lineage per temperature. *r* = minimum correlation coefficient for wing-leg relationship per lineage per temperature.
[2] Mean change in ILSR slope from 17 ˚C to 25 ˚C, across 1000 bootstrap replicates. *P*-value shown in parenthesis.
[3] Mean change in ILSR slope from 25 ˚C to 28 ˚C, across 1000 bootstrap replicates. *P*-value shown in parenthesis.
